# Anti-Inflammatory Effects of 6,8-Diprenyl-7,4′-dihydroxyflavanone from *Sophora tonkinensis* on Lipopolysaccharide-Stimulated RAW 264.7 Cells

**DOI:** 10.3390/molecules21081049

**Published:** 2016-08-11

**Authors:** Hee-Sung Chae, Hunseung Yoo, Young-Mi Kim, Young Hee Choi, Chang Hoon Lee, Young-Won Chin

**Affiliations:** 1 College of Pharmacy and Integrated Research Institute for Drug Development, Dongguk University-Seoul, 32 Dongguk-lo, Ilsandong-gu, Goyang-si, Gyeonggi-do 410-820, Korea; chaeheesung83@gmail.com (H.-S.C.); 0210121@hanmail.net (Y.-M.K.); choiyh@dongguk.edu (Y.H.C.); uatheone@dongguk.edu (C.H.L.); 2 New Drug Preclinical & Analytical Team, Life Science R & D Center, SK Chemicals, 310 Pangyo-ro 463-400, Korea; yoohoo0733@naver.com

**Keywords:** 6,8-diprenyl-7,4′-dihydroxyflavanone, anti-inflammatory, nuclear factor-κB, extracellular signal-regulated kinases

## Abstract

The anti-inflammatory effects and molecular mechanism of 6,8-diprenyl-7,4′-dihydroxyflavanone (DDF), one of the flavanones found in *Sophora tonkinensis*, were assessed in vitro through macrophage-mediated inflammation in the present study. The anti-inflammatory effects of DDF were not previously reported. DDF inhibited the production of nitric oxide and the expression of tumor necrosis factor α, interleukin-1β, and interleukin-6. Furthermore, the activation of nuclear factor-κB (NF-κB) and extracellular signal-regulated kinases (ERKs) in lipopolysaccharide-stimulated macrophages was suppressed by treatment with DDF. Therefore, DDF demonstrated potentially anti-inflammatory effects via the blockade of NF-κB and ERK activation in macrophages.

## 1. Introduction

Inflammation is a normal reaction to control foreign factors or injury and is associated with the tissue influx of large numbers of immune cells [1,2,3,4]. Of the immune cells, macrophages are long-lived tissue cell types with the capacity to produce a wide array of biological mediators involved in immune responses [5]. Macrophages display biologically diverse functions, including immune stimulatory or immune suppressive effects and promotion or restraint of inflammation [6,7]. In particular, during the inflammation process, mitogen-activated protein kinases (MAPKs) and nuclear factor-kappa B (NF-κB) are known to be involved in producing inflammatory mediators in macrophages [8]. The activation of MAPKs phosphorylates and activates other kinases or transcription factors, thereby altering the expression of inflammatory genes [9]. Additionally, nuclear factor-kappa B (NF-κB) regulates the expression of genes relevant in controlling cell proliferation, inflammatory responses, and cell adhesion [10]. In light of the roles of MAPKs and NF-κB as regulators in the expression of genes involved in inflammatory and immune reactions, MAPKs and NF-κB have gained much attention as attractive therapeutic targets in the discovery of anti-inflammatory agents [11].

**Figure 1:**
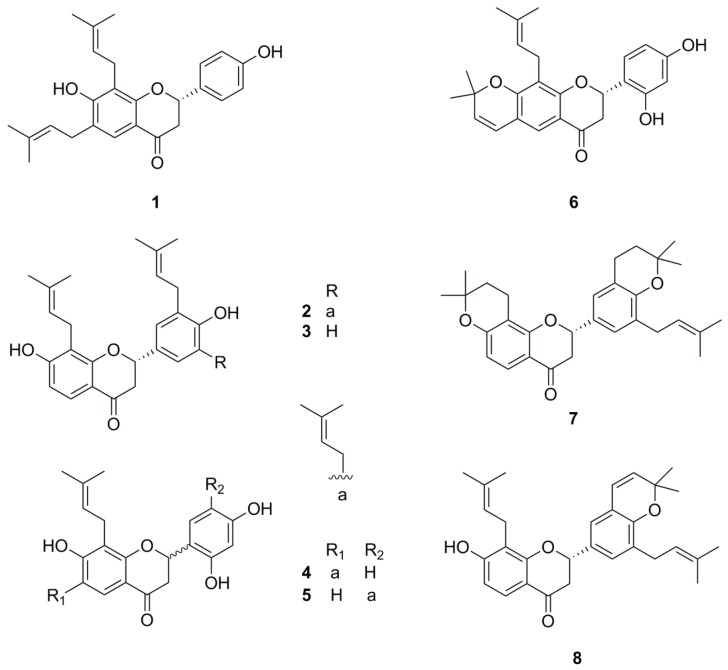
Chemical structures of compounds isolated from *S. tonkinensis*.

**Figure 2:**
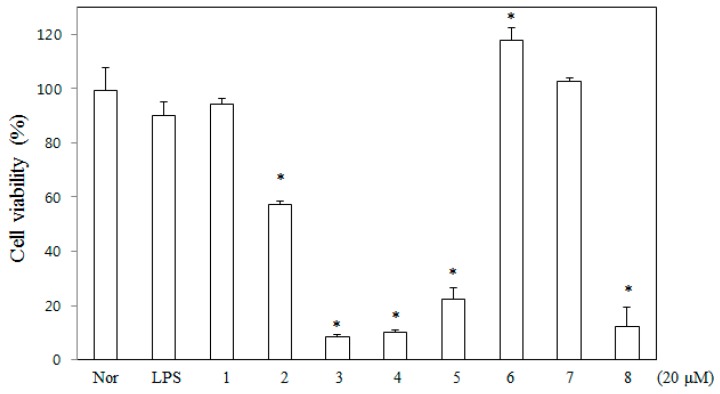
Effects of eight flavanones on the viability of RAW 264.7 cells. Cells grown in serum-free media were treated with 20 μM of each compound for 24 h, and cell viability was assessed by MTT assay, as described in the Materials and Methods section. Results of independent experiments were averaged and are shown as the percentage of cell viability compared with the viability of normal control cells. Nor (untreated control cells); LPS (lipopolysaccharide-treated cells); 6,8-diprenyl-7,4′-dihydroxyflavanone (**1**); sophoranone (**2**); glabrol (**3**); 6,8-diprenyl-7,2′,4′-trihydroxyflavanone (**4**); 8,5′-diprenyl-7,2′,4′-trihydroxyflavanone (**5**); flemichin (**6**); tonkinochromane A (**7**); sophoranochromene (**8**). Data represent the mean ± SEM of three experiments. * Significant difference from Nor, *p* < 0.05.

**Figure 3:**
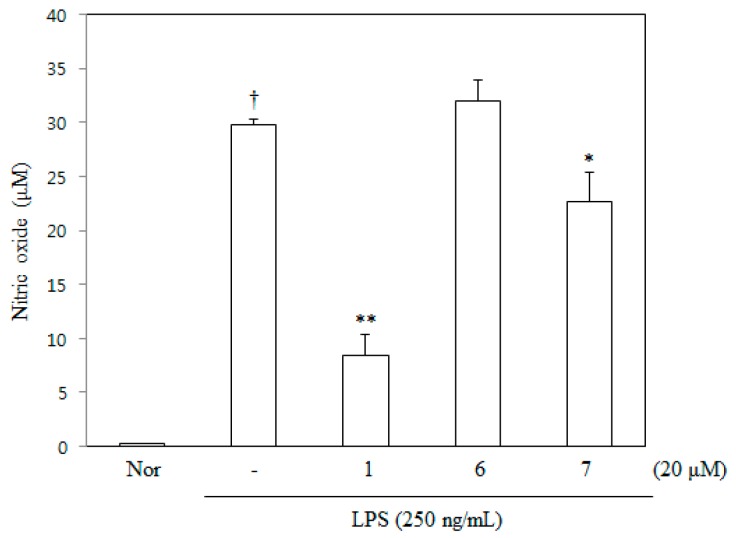
Effects of three non-toxic flavanones on the inhibition of LPS-stimulated NO production in RAW 264.7 cells. Cells cultured in phenol red- and serum-free media were pretreated with each compound for 1 h and then stimulated with 250 ng/mL final concentration LPS for 24 h. In the culture medium, NO production was measured based on the Griess reaction, as described in the Materials and Methods section. Nor (untreated control cells); 6,8-diprenyl-7,4′-dihydroxyflavanone (**1**); flemichin (**6**); tonkinochromane A (**7**). Values are expressed as the mean ± SEM. † Significant difference from normal cells, * significant difference from LPS cells, *p* < 0.05 and ** significant difference from LPS cells, *p* < 0.01.

As part of our ongoing search to discover anti-inflammatory agents from medicinal plants [12,13], 6,8-diprenyl-7,4′-dihydroxyflavanone (DDF) was newly isolated from the roots of *Sophora tonkinensis*. Seven flavanones were isolated as chemical constituents from the roots of *Sophora tonkinensis* previously, and these compounds were used in the present study [12]. Flavanones from *Sophora tonkinensis* were tested for their inhibitory activity against nitric oxide (NO) production in lipopolysaccharide (LPS)-stimulated RAW 264.7 cells, since previous studies reported on the modulation of inflammatory cytokines [14,15,16]. Of the tested compounds (Figure 1), DDF was found to significantly inhibit NO production without cytotoxicity (Figure 2 and Figure 3). However, the anti-inflammatory mechanism of DDF on macrophages remains unknown. In the present study, DDF was assessed for its inhibitory effect on inflammatory mediator production, mRNA expression of cytokines, and activation of NF-κB and MAPKs in LPS-stimulated RAW 264.7 cells.

## 2. Results

### 2.1. Toxicity of DDF on Macrophages

To determine the effect of DDF on cell viability, various concentrations of DDF were tested with MTT assay using RAW 264.7 cells grown in serum-free media. Eight flavanones, DDF, sophoranone (**2**), glabrol (**3**), 6,8-diprenyl-7,2′,4′-trihydroxyflavanone (**4**), 8,5′-diprenyl-7,2′,4′-trihydroxyflavanone (**5**), flemichin D (**6**), tonkinochromane A (**7**), and sophoranochromene (**8**) were tested for their toxic effect, and the results are shown in Figure 2. Neither DDF nor dimethyl sulfoxide (DMSO) exerted any significant toxic effect on RAW 264.7 cells under the tested concentrations after 24 h of treatment. Therefore, non-toxic concentrations of DDF were used in all the experiments.

### 2.2. NO Inhibitory Activity of DDF on RAW 264.7 Cells

Three flavanones of *Sophora tonkinensis* were selected from the cytotoxicity test. DDF, flemichin D, and tonkinochromane A were evaluated for their inhibitory activity against NO production in LPS-stimulated RAW 264.7 cells. The effect of DDF on LPS-stimulated NO production in RAW 264.7 cells was investigated by measuring the amount of nitrite released into the culture medium based on the Griess reaction. DDF suppressed nitrite production in a concentration-dependent manner with an IC50 value of 12.21 μM (Figure 3).

### 2.3. Effect of DDF on iNOS and COX-2 Expression Levels in RAW 264.7 Cells

**Figure 4:**
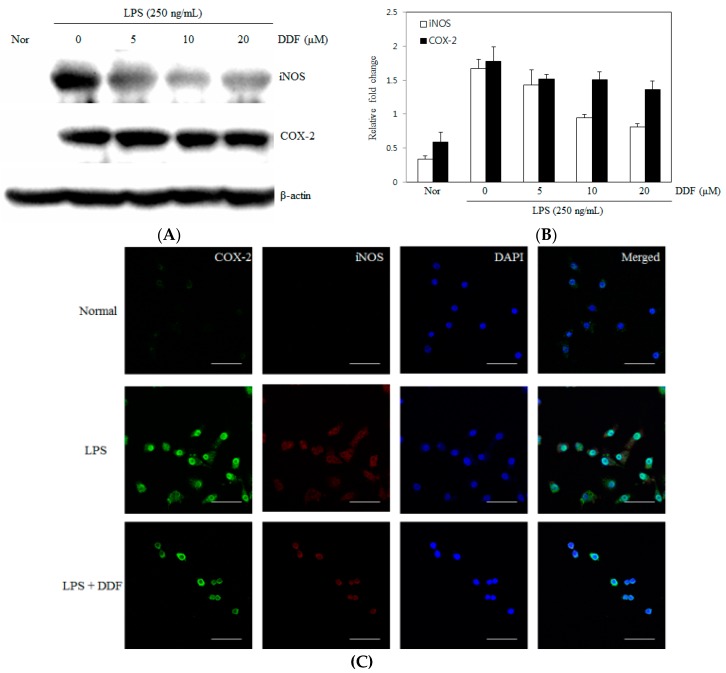
Inhibition of inducible nitric oxide synthase (iNOS) protein expression by DDF. (**A**) RAW 264.7 cells were pretreated with different concentrations of DDF for 30 min and stimulated with LPS (250 ng/mL) for a further 24 h. Equal amounts of proteins in the cell lysates were separated by electrophoresis, and the protein expression levels of iNOS and COX-2 were determined with specific antibodies against iNOS and COX-2; (**B**) Immunoblot signals were quantified using Molecular Analyst/PC densitometry software (Version 4.6, Bio-Rad, Hercules, CA, USA), and densitometric analysis is reported. The contents of iNOS and COX-2 in the cell lysate were normalized to the content of β-actin; (**C**) Expression of iNOS and COX-2 with DDF. Cells were cultured for 24 h with LPS (250 ng/mL), fixed, permeabilized, and incubated with rabbit polyclonal anti-iNOS antibody, followed by Alexa-488-conjugated anti-rabbit Ig (green), and with mouse polyclonal anti-COX-2 antibody followed by Alexa-594-conjugated anti-mouse Ig (red). The nuclei of the corresponding cells were visualized by DAPI staining (blue). Normal, untreated control cells; LPS, treatment with only LPS (250 ng/mL); DDF, 6.8-diprenyl-4′,7-dihydroxyflavanone; (magnification: 60×, scale bars: 50 μm).

Nitric oxide synthase (iNOS) and cyclooxygenase-2 (COX-2) are the inflammatory factors associated with LPS stimulation. To investigate the anti-inflammatory activity of DDF, we tested the effects of DDF on LPS-stimulated iNOS and COX-2 upregulation in RAW 264.7 cells by western blot analysis and immunofluorescence. As shown in Figure 4A, iNOS and COX-2 protein expressions were not detected in unstimulated cells; however, expression levels were markedly increased 24 h after stimulation with 250 ng/mL LPS. Cells pretreated with DDF (5–20 μM) showed a concentration-dependent inhibition of iNOS protein expression but had a weak effect on the expression of COX-2 following LPS stimulation for 24 h. As shown by immunofluorescence analysis in Figure 4C, iNOS and COX-2 were not detected in normal control cells; however, their levels markedly increased after treatment with 250 ng/mL LPS for 24 h. Cells pretreated with DDF inhibited iNOS expression but had a weak effect on the expression of COX-2 following LPS stimulation for 24 h.

### 2.4. Activation of the MAPK Pathway by DDF

**Figure 5:**
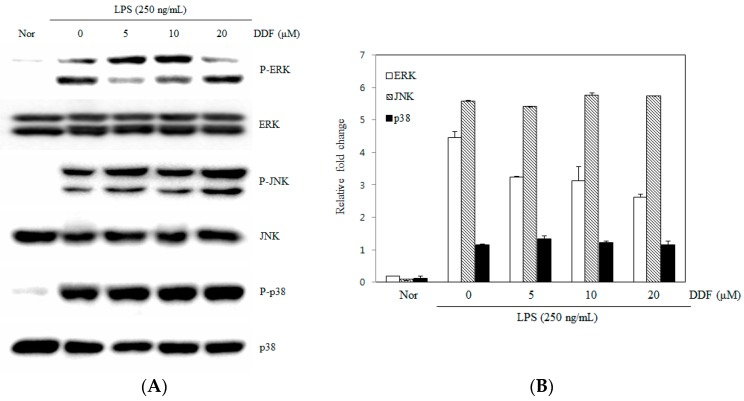
Effect of DDF on the phosphorylation of MAPKs in LPS-stimulated RAW 264.7 cells. Cells were treated with the indicated concentrations of DDF for 30 min prior to incubation with LPS (250 ng/mL) for a further 30 min. (**A**) Whole cell lysates were subsequently analyzed by immunoblotting; (**B**) Immunoblot signals were quantified using Molecular Analyst/PC densitometry software (Version 4.6, Bio-Rad). Densitometric analysis of phosphorylated isoforms is reported. The contents of ERK, JNK, and p38 in the cell lysate were normalized to the content of total form.

In order to elucidate the underlying mechanisms of DDF, we examined the effects of DDF on the activation of MAPKs. The stimulation of RAW 264.7 cells with LPS resulted in an increased phosphorylation of all three types of MAPKs (p38, JNK, and ERK) 30 min post-treatment. DDF suppressed the phosphorylation of ERK1/2 but had no effect on the phosphorylation of JNK1/2 or p38 MAPK (Figure 5).

### 2.5. Modulation of the NF-κB Pathway by DDF

**Figure 6:**
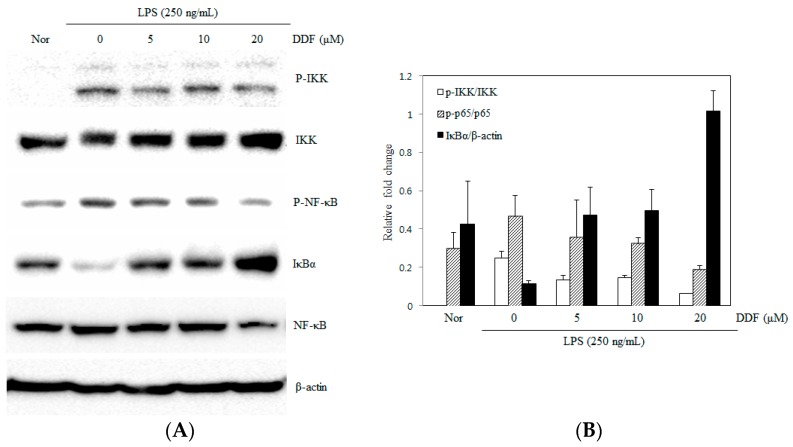
Effect of DDF on IκBα degradation and phosphorylation of IKK and NF-κB (p65) in LPS-stimulated RAW 264.7 cells. (**A**) Cells were pretreated with three different concentrations of DDF for 30 min. To evaluate the levels of p-IKK, IKK, IκBα, p-NF-κB, and NF-κB, the cells were stimulated with LPS for 30 min, after which the cell lysates were analyzed by immnoblot analysis; (**B**) Immunoblot signals were quantified using Molecular Analyst/PC densitometry software (Version 4.6, Bio-Rad).

To evaluate the mechanisms of DDF that affect the gene expression of pro-inflammatory cytokines, we examined the effects of DDF on NF-κB activation [17]. Stimulation of RAW 264.7 cells with LPS induced the degradation of IκBα and activation of NF-κB after 0.5 h of incubation. The phosphorylation of IKK and NF-κB (p65) was significantly reduced and almost abolished by pretreatment with 20 μM DDF, as shown by western blot analysis (Figure 6). In addition, DDF inhibited the induced degradation of IκBα, thereby preventing the expression of NF-κB-regulated genes.

### 2.6. Effect of DDF on TNF-α, IL-1β, and IL-6

**Figure 7:**
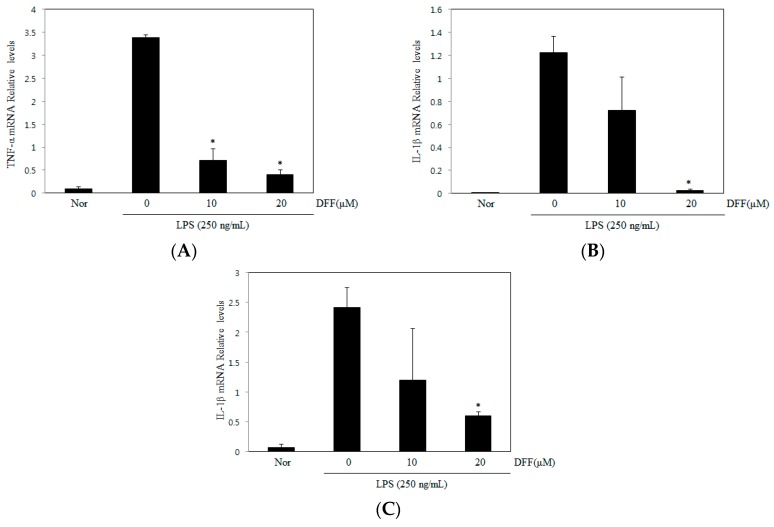
Effect of DDF on macrophage cytokines. LPS-stimulated macrophages were cultured in the presence or absence of DDF. Cytokine levels of (**A**) tumor necrosis factor-α (TNF-α); (**B**) interleukin-1β (IL-1β); and (**C**) interleukin-6 (IL-6) were measured by qRT-PCR. Nor, untreated control cells; LPS, treatment with only LPS (250 ng/mL). Data represent the mean ± SEM of three experiments. * Significant difference from LPS cells, *p* < 0.05.

NF-κB and MAPKs act as the major regulators of the expression of cytokine genes following LPS stimulation. We examined the effect of DDF on the expression of TNF-α, IL-1β, and IL-6 mRNA in macrophages. As shown in Figure 7, pretreatment with DDF significantly lowered the expression of TNF-α, IL-1β, and IL-6 in LPS-stimulated RAW 264.7 cells.

## 3. Discussion

Nuclear factor-κB (NF-κB) is an important transcription factor that controls the gene expression of cytokines, chemokines, and growth factors in both normal and abnormal states [18]. The activation of NF-κB subsequently promotes the transcription of a number of genes involved in inflammation, including inducible iNOS and COX-2 [19]. Inappropriate activation of NF-κB or MAPK has been shown to be associated with the pathophysiological mechanisms of cancer and inflammation [20]. Therefore, the discovery of active compounds from natural products that can regulate the activation of NF-κB and MAPKs may lead to the development of anti-inflammatory therapeutics. Since ERK, JNK, and p38 MAPKs are known to be associated with LPS-mediated induction of iNOS and COX-2 in macrophages [21,22], we investigated the effect of DDF on the activation of MAPKs in LPS-stimulated macrophages. When cells were pretreated with DDF and LPS for 30 min, DDF was found to attenuate the LPS-stimulated activation of ERK but did not suppress either the expression of JNK or p38 MAPK (Figure 5). The expression of a number of inflammatory genes is regulated through the NF-κB pathway [23]. Under normal conditions, the complex form of NF-κB and IκBα is predominantly localized in the cytosol. During the inflammation process in response to various stimuli, IκB has to be phosphorylated by IκB kinases (IKK) to release NF-κB (p65) and facilitate NF-κB translocation into the nucleus; concomitantly, the IκB subunit is degraded by proteasomes [24,25]. In our study, we examined NF-κB and IKK phosphorylation as well as the degradation of IκBα. Immunoblot/western blot analysis revealed that the inhibition of NF-κB activity by DDF may have resulted from the inhibition of IκBα degradation (Figure 6).

Inducible iNOS expression is induced by LPS or cytokines in the immune cells, catalyzing the oxidative deamination of L-arginine to produce NO, a potential immune mediator [26]. Overproduction of NO appears to be connected to tissue damage and organ dysfunction [27]. In the present study, we demonstrated that DDF inhibited LPS-stimulated NO production in RAW 264.7 cells. Our results showed that DDF inhibition of NO production in LPS-stimulated RAW 264.7 cells occurred via the inhibition of iNOS protein expression in a dose-dependent manner. 

In conclusion, our results demonstrated that DDF regulated the expression of nitric oxide, TNF-α, IL-1β, and IL-6 mRNA in LPS-stimulated macrophages. DDF also decreased iNOS expression. Moreover, DDF suppressed the ERK1/2 and NF-κB pathways. Therefore, DDF can exert anti-inflammatory effects via the inhibition of NF-κB and ERK activation in macrophages.

## 4. Materials and Methods 

### 4.1. Preparation of Compound ***1***

About 400 g of extract were obtained from 10 kg of *S. tonkinensis* root. The solid extract was then subjected to column chromatography (CC) over Diaion HP-20 eluted with MeOH/H_2_O (50%, 70%, 90%, 100%), and then with acetone to produce five fractions (ST1–ST5). The ST4 fraction (30 g) was subjected to silica gel CC with gradient mixtures of CHCl_3_ and MeOH (50:1, 30:1, 15:1, 10:1, 8:1, 5:1, 1:1, 0:1, 2 L each) to produce 22 sub-fractions (ST401–ST422). A combination of ST403 and ST404 (ST4C) was purified by MPLC (Biotage) and eluted with gradient mixtures of MeOH and H_2_O (20:80–90:10), which provided 26 sub-fractions (ST4C01–ST4C26). Compound **1** (DDF, 6.5 mg) was precipitated from ST4C13. The structure of compound **1** was confirmed by comparing NMR data with those reported in the literature [28]. The detail data were shown in Supplementary Materials.

### 4.2. Cell Culture

Murine macrophages, RAW 264.7, were obtained from the Korean Research Institute of Bioscience and Biotechnology (Daejeon, Korea) and grown in RPMI medium containing 10% fetal bovine serum and 100 U/mL penicillin/streptomycin sulfate. Cells were incubated in a humidified 5% CO_2_ atmosphere at 37 °C.

### 4.3. Drugs and Chemicals

RPMI, penicillin, and streptomycin were purchased from Hyclone (Logan, UT, USA). Bovine serum albumin and LPS were purchased from Sigma (St. Louis, MO, USA). Antibodies against iNOS, COX-2, p-IKK, IKK, IκBα, p-p65 (NF-κB), p65 (NF-κB), β-actin, p-ERK, ERK, p-JNK, JNK, p-p38, and p38 were purchased from Cell Signaling Technology, Inc. (Danvers, MA, USA). Oligonucleotide primers for TNF-α, IL-6, IL-1β, and GAPDH were purchased from Bioneer Corp. (Daejeon, Korea).

### 4.4. MTT Assay for Cell Viability

Cells were seeded into 96-well plates at a density of 5 × 10^4^ cells/well and incubated with serum-free media in the presence of different concentrations of DDF. Following incubation for 24 h, 10 μL of 3-(4,5-dimethylthiazol-2-yl)-2,5-diphenyl tetrazolium bromide (MTT) (5 mg/mL in saline) was added and incubated for another 4 h. MTT was converted by mitochondrial succinate dehydrogenase in live cells into visible formazan crystals during incubation. The formazan crystals were then solubilized in DMSO, and the absorbance was measured at 540 nm using an enzyme-linked immunosorbent assay (ELISA) microplate reader (Benchmark, Bio-Rad Laboratories). Relative cell viability was calculated by comparison with the absorbance of the untreated control group. All experiments were performed in triplicate.

### 4.5. Measurement of NO Production

Nitrite concentration in the culture medium was measured as an indicator of NO production based on the Griess reaction [29]. RAW 264.7 cells (2 × 10^5^ cells/well) were cultured in 96-well plates using RPMI without phenol red, and cells were pretreated with different concentrations of DDF for 1 h. Cellular NO production was induced by the addition of 250 ng/mL final concentration LPS and incubation for 24 h. Following incubation, 100 μL conditioned media was mixed with the same volume of Griess reagent and incubated for 15 min. The absorbance of the mixture at 540 nm was measured with an ELISA microplate reader (Benchmark, Bio-Rad Laboratories).

### 4.6. Immunoblot/Western Blot Analysis

Protein expression was assessed by western blot analysis according to standard procedures. RAW 264.7 cells were cultured in 60-mm culture dishes (2 × 10^6^/mL) and pretreated with various concentrations of DDF (5, 10, and 20 μM). After 30 min of pretreatment, LPS was added to the culture medium, and the cells were incubated at 37 °C for 30 min. Following incubation, the cells were washed twice with ice-cold PBS (pH 7.4). The DDF-treated cell pellets were then resuspended in lysis buffer for 15 min on ice, after which cell debris was removed by centrifugation. Immunoreactive bands were developed using the chemiluminescent substrate ECL Plus (Thermo Fisher Scientific, Pittsburgh, PA, USA).

### 4.7. Quantitative Real-Time RT-PCR

Total cellular RNA was isolated using a TRIzol RNA extraction kit according to the manufacturer’s instructions. Briefly, total RNA (1 μg) was converted to cDNA by treatment with 200 units of reverse transcriptase and 500 ng oligo-dT primer in 50 mM Tris-HCl (pH 8.3), 75 mM KCl, 3 mM MgCl_2_, 10 mM DTT, and 1 mM dNTPs at 42 °C for 1 h. The reaction was then stopped by incubating the solution at 70 °C for 15 min, after which 1 μL cDNA mixture was used for enzymatic amplification. PCR reactions were performed using 1 μL cDNA and 9 μL master mix, containing iQ SYBR Green Supermix (Bio-Rad), 5 pmol forward primer, and 5 pmol reverse primer with a CFX384 Real-Time PCR Detection System (Bio-Rad) as follows: 3 min at 95 °C followed by 40 cycles of 10 s at 95 °C and 30 s at 55 °C. The fluorescence signal generated from SYBR Green I DNA dye was measured during the annealing steps. Specificity of the amplification was confirmed by melting curve analysis. Data were collected and recorded by CFX Manager Software (Bio-Rad) and expressed as a function of threshold cycle (C_T_). The relative quantity of the gene of interest was then normalized to the relative quantity of hypoxanthine phosphoribosyltransferase (ΔΔCT). The sample mRNA abundance was calculated by the equation 2^−(ΔΔCT)^. The specific primer sets used were (5′ to 3′): IL-6 CAAAGCCAGAGTCCTTCAGAG (forward), GCCACTCCTTCTGTGACTCC (reverse); GAPDH TGTTCCTACCCCCAATGTGT (forward), TGTGAGGGAGATGCTCAGTG (reverse); IL1β GACCTTCCAGGATGAGGACA (forward), TGTTCATCTCGGAGCCTGTA (reverse); TNF-α CAAATGGCCTCCCTCTCAT (forward), TGGGCTACAGGCTTGTCACT (reverse). Gene-specific primers were custom-synthesized by and purchased from Bioneer (Daejeon, Korea).

### 4.8. Immunofluorescence

RAW 264.7 cells cultured on Permanox plastic chamber slides were fixed with ethanol for 30 min at 4 °C. After washing with PBS and blocking with 3% bovine serum albumin in PBS for 30 min, samples were incubated overnight at 4 °C with rabbit monoclonal anti-COX2 and anti-iNOS (1:200 dilutions, Abcam, Cambridge, MA, USA). The excess primary antibodies were then removed, slides were washed with PBS, and the samples were incubated with Alexa 488-conjugated and Alexa 594-conjugated secondary antibodies (Invitrogen Molecular Probes, Burlington, ON, Canada) for 2 h at room temperature. After washing with PBS, slides were mounted using ProLong Gold Antifade reagent containing 4′,6-diamidino-2-phenylindole (DAPI) (Thermo Fisher Scientific) to visualize the nuclei. Specimens were covered with coverslips and evaluated under a confocal laser scanning microscope (Nikon Eclipse, Nikon, Japan).

### 4.9. Statistical Analysis

Experimental data are presented as the mean ± SEM. The level of statistical significance was determined by analysis of variance (ANOVA) followed by Dunnett’s *t*-test for multiple comparisons. *p* Values less than 0.05 were considered significant.

## Supplementary Materials

Supplementary materials can be accessed at: http://www.mdpi.com/1420-3049/21/8/1049/s1.

## Abbreviations

The following abbreviations are used in this manuscript:

DDF	6,8-diprenyl-7,4′-dihydroxyflavanone
So	sophoranone
Gl	glabrol
5DTF	8,5′-diprenyl-7,2′,4′-trihydroxyflavanone
Fl	flemichin D
To	tonkinochromane A
SC	sophoranochromene
iNOS	inducible nitric oxide synthases
MAPKs	mitogen-activated protein kinases
NF-κB	nuclear factor-kappa B
