# Correction: Chae, H.S., et al. Anti-Inflammatory Effects of 6,8-Diprenyl-7,4′-dihydroxyflavanone from *Sophora tonkinensis* on Lipopolysaccharide-Stimulated RAW 264.7 Cells. *Molecules*. 2016, 21, 1049.

**DOI:** 10.3390/molecules21101413

**Published:** 2016-10-24

**Authors:** Hee-Sung Chae, Hunseung Yoo, Young-Mi Kim, Young Hee Choi, Chang Hoon Lee, Young-Won Chin

**Affiliations:** 1 College of Pharmacy, Dongguk University-Seoul, 32 Dongguk-lo, Ilsandong-gu, Goyang-si, Gyeonggi-do 410-820, Korea; chaeheesung83@gmail.com (H.-S.C.); 0210121@hanmail.net (Y.-M.K.); choiyh@dongguk.edu (Y.H.C.); uatheone@dongguk.edu (C.H.L.); 2 New Drug Preclinical & Analytical Team, Life Science R & D Center, SK Chemicals, 310 Pangyo-ro 463-400, Korea; yoohoo0733@naver.com

The authors wish to make the following correction to their paper [1]. In Panel C of Figure 4, the data was incorrectly displayed. The correct version of Figure 4C is as follows:

The change does not affect the scientific results. The manuscript will be updated and the original will remain online on the article webpage. The authors would like to apologize for any inconvenience caused to readers by these changes.

## Figures and Tables

**Figure 4:**
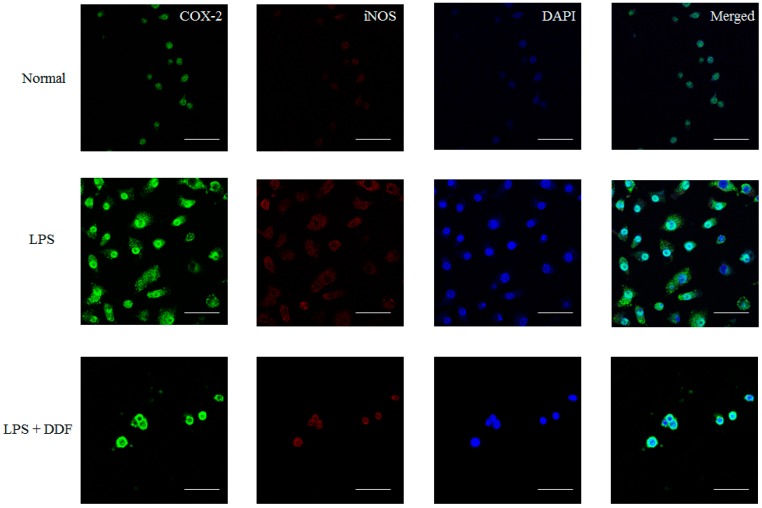
(**C**) Expression of iNOS and COX-2 with DDF. Cells were cultured for 24 h with LPS (250 ng/mL), fixed, permeabilized, and incubated with rabbit polyclonal anti-iNOS antibody, followed by Alexa-488-conjugated anti-rabbit Ig (green); and with mouse polyclonal anti-COX-2 antibody followed by Alexa-594-conjugated anti-mouse Ig (red); The nuclei of the corresponding cells were visualized by DAPI staining (blue). Normal, untreated control cells; LPS, treatment with only LPS (250 ng/mL); DDF, 6.8-diprenyl-4′,7-dihydroxyflavanone; (magnification: 60×, scale bars: 50 μm).
