# Supplementary material for: Anti-Inflammatory Effects of 6,8-Diprenyl-7,4′-dihydroxyflavanone from Sophora tonkinensis on Lipopolysaccharide-Stimulated RAW 264.7 Cells
Source: Molecules. 2016 Aug 11;21(8):1049. doi: 10.3390/molecules21081049 (PMC6274169; doi:10.3390/molecules21081049)
Supplement: Supplementary file 1 [file molecules-21-01049-s001.pdf]

Supplementary Materials: Anti-Inflammatory Effects of 6,8-Diprenyl-7,4′-dihydroxyflavanone from *Sophora tonkinensis* in Lipopolysaccharide-Stimulated RAW 264.7 Cells

Hee-Sung Chae, Hunseung Yoo, Young-Mi Kim, Young Hee Choi, Chang Hoon Lee and Young-Won Chin

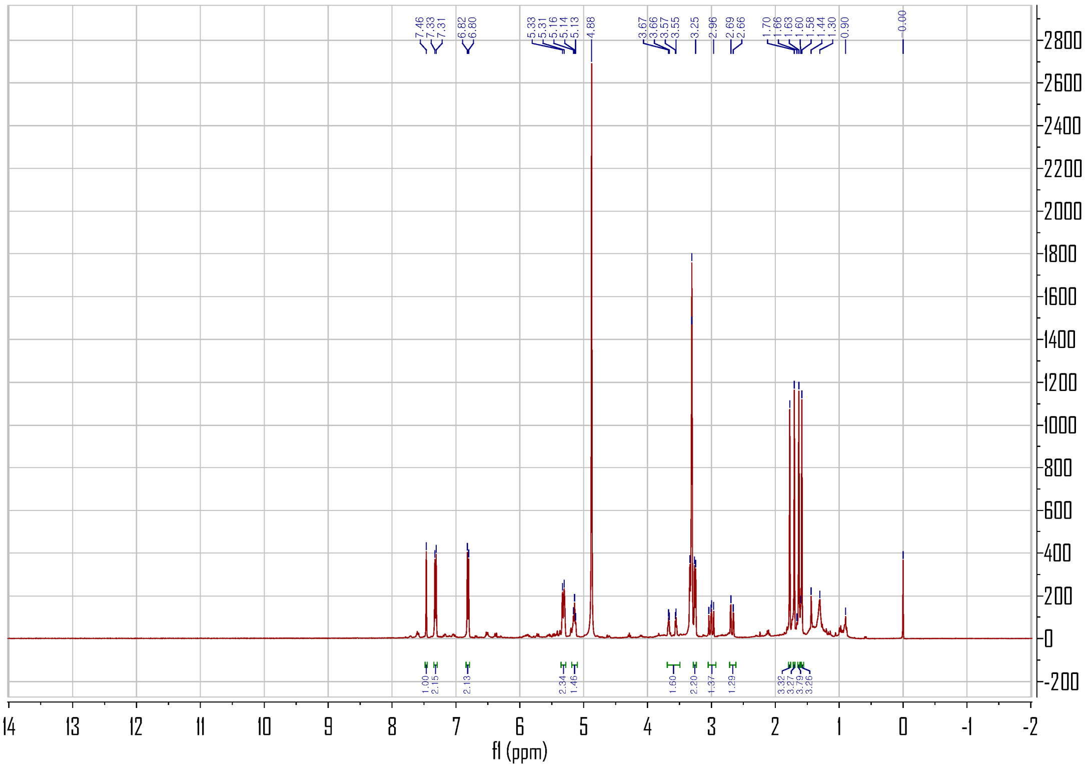

Figure S1. ^{1}H-NMR spectrum of 6,8-diprenyl-7,4′-dihydroxyflavanone.

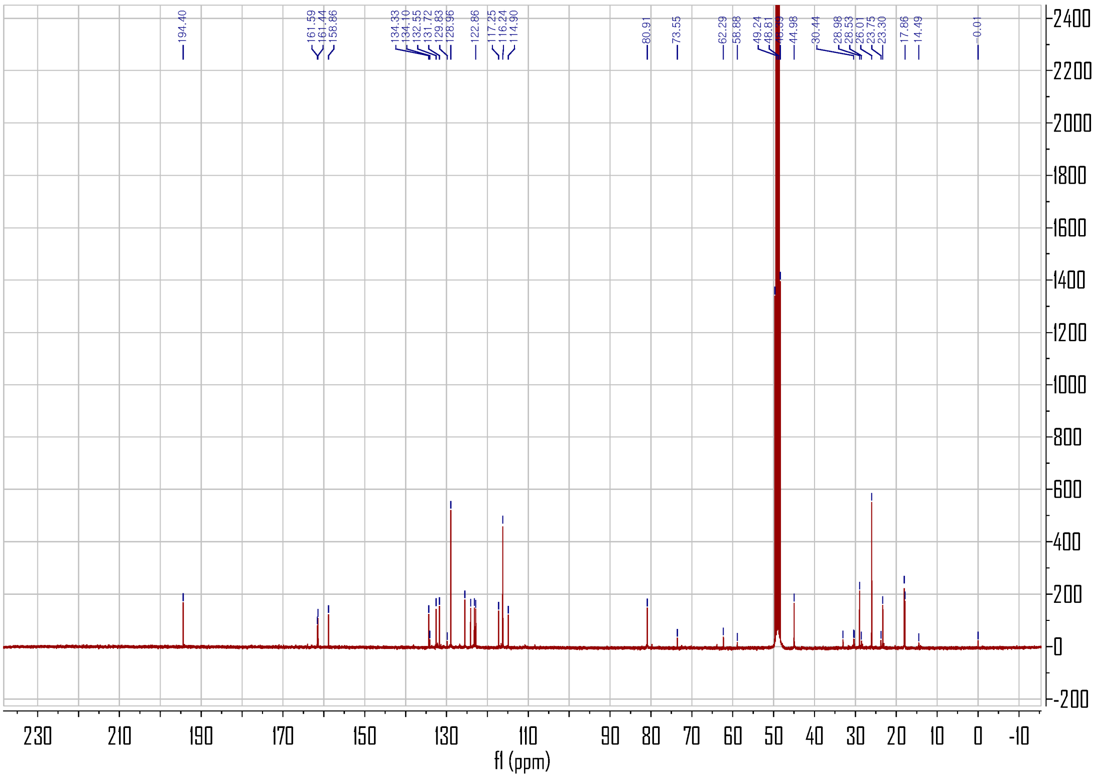

Figure S2. ^{13}C-NMR spectrum of 6,8-diprenyl-7,4′-dihydroxyflavanone.
